# Peripheral Nerve Stimulation for Perioperative Care in Oncologic Surgical Cases: A Narrative Review

**DOI:** 10.3390/healthcare14121767

**Published:** 2026-06-19

**Authors:** Taylor Johnson, Jeremy Ashton Hunter Boyd, Sreyansh Rishabh, Sanjib Adhikary

**Affiliations:** 1 Department of Anesthesiology and Perioperative Medicine, Penn State Health Milton S. Hershey Medical Center, 500 University Drive, Hershey, PA 17033, USA; tjohnson11@pennstatehealth.psu.edu; 2 Penn State Hershey College of Medicine, Penn State Health Milton S. Hershey Medical Center, 500 University Drive, Hershey, PA 17033, USA; jboyd8@pennstatehealth.psu.edu; 3 Department of Kinesiology, Pennsylvania State University at Harrisburg, 777 West Harrisburg Pike, Middletown, PA 17057, USA; srishabh@pennstatehealth.psu.edu

**Keywords:** peripheral nerve stimulation, cancer pain, oncologic surgery, opioid-sparing, neuromodulation, chemotherapy-induced peripheral neuropathy, ERAS, perioperative analgesia, narrative review, SANRA

## Abstract

**Highlights:**

**What are the main findings?**

- Peripheral nerve stimulation (PNS) is feasible across diverse oncologic pain subtypes, including post-thoracotomy pain, post-mastectomy pain syndrome, brachial plexopathy, and chemotherapy-induced peripheral neuropathy, with a 58–67% success rate in cancer-specific series and opioid consumption reductions of up to 80–90% in the PAINfRE randomized controlled trial of an analogous surgical population.
- Current oncologic-specific evidence is predominantly Level 4 (pilot and retrospective series); Level 1–2 evidence from non-cancer randomized controlled trials and systematic reviews provides the mechanistic and efficacy foundation supporting extrapolation to perioperative cancer care.

**What are the implications of the main findings?**

- The opioid-sparing properties of minimally invasive PNS systems carry immunological significance in cancer surgery: reducing perioperative opioid exposure may help preserve natural killer cell function and antitumor immune surveillance during the critical postoperative window when circulating tumor cells are at greatest risk of metastatic seeding.
- Adequately powered, oncologic-specific randomized controlled trials are needed to establish evidence-based recommendations; in the interim, PNS is a viable multimodal analgesic adjunct for integration into enhanced recovery after surgery (ERAS) protocols for cancer surgery, particularly for thoracic and breast oncology where chronic post-surgical pain syndromes are prevalent.

**Abstract:**

**Background:** Cancer pain affects approximately 44.5% of all patients with malignancy and up to 55–65% of those with advanced or metastatic disease; a substantial proportion remain inadequately controlled with conventional pharmacological approaches alone. Peripheral nerve stimulation (PNS), a minimally invasive neuromodulatory strategy, has emerged as a potential opioid-sparing analgesic option for the perioperative management of oncologic surgical patients. **Objectives:** This narrative review synthesizes current evidence on the application, mechanisms, clinical efficacy, safety, and integration of temporary and permanent PNS systems in cancer patients, with specific focus on cancer-specific pain syndromes, key clinical studies, opioid-sparing immunological implications, evidence quality, and directions for future research. **Methods:** As a narrative review, this work was structured in accordance with the Scale for the Assessment of Narrative Review Articles (SANRA) to ensure methodological transparency. A focused, non-systematic literature search of PubMed/MEDLINE, Embase, and the Cochrane Library was performed from database inception through March 2026, supplemented by hand-searching of reference lists and targeted retrieval of clinical practice guidelines. Sources were selected on the basis of relevance to PNS or closely analogous peripheral neurostimulation modalities in oncologic, perioperative, or chronic pain contexts. Evidence was synthesized narratively, with each cited study graded using the Oxford Centre for Evidence-Based Medicine (OCEBM) 2011 Levels of Evidence framework to enable transparent calibration of confidence. **Results:** Available preliminary and largely extrapolated evidence supports PNS as a promising but not yet established useful adjunct in oncologic perioperative care; because cancer-specific data rest substantially on a single pilot study (n = 12), one retrospective review (n = 15), and extrapolation from non-cancer populations, these conclusions should be regarded as hypothesis-generating. Randomized controlled trial data from non-cancer cohorts demonstrate opioid consumption reductions of approximately 80–90% in the PAINfRE trial, while the post-amputation trial demonstrated ≥50% pain-relief responder rates and reductions in pain interference, with clinically meaningful improvements in pain and function. Oncologic-specific pilot and retrospective evidence confirms feasibility and a 58–67% success rate across diverse cancer pain subtypes. **Conclusions:** The opioid-sparing properties of PNS carry additional biological plausibility for preserving perioperative antitumor immune function. High-quality prospective trials specifically designed for oncologic surgical populations remain needed to establish evidence-based recommendations.

## 1. Introduction

### 1.1. The Scope and Justification for the Review

Cancer pain remains among the most undertreated conditions in modern medicine. A 2023 systematic review reported an overall pain prevalence of 44.5% across all cancer patients, with moderate-to-severe pain affecting 30.6%. A 2026 meta-analysis encompassing 36 studies (n = 39,806) estimated a pooled chronic pain prevalence of 41% among cancer survivors, with 40.2% receiving insufficient pharmacological pain control. Prevalence is strongly stage-dependent—approximately 50% in early-stage disease and ≥75% in advanced or metastatic malignancy—and 10–15% of patients develop severe or intractable pain refractory to standard pharmacological regimens, with neuropathic pain patients approximately 2.6 times more likely to experience inadequate relief than those with purely nociceptive presentations [1] (p. 591), [2] (pp. 118–125), [3] (pp. 489–501), [4] (p. 510), [5].

Neuropathic cancer pain—arising from tumor invasion or compression of neural structures, treatment-induced nerve injury, or metabolic dysfunction—affects up to 40% of cancer patients and poses particular treatment challenges owing to diminished opioid responsiveness. Cancer pain may originate from direct tumor effects, treatment-related sequelae (surgery, chemotherapy, radiation, and immunotherapy), cancer-associated conditions, and comorbid pain conditions. Because the global cancer incidence is projected to increase by 77% between 2022 and 2050, the burden of cancer-related pain will intensify proportionally, justifying the importance of developing and validating a diversified analgesic armamentarium [2] (pp. 118–125), [5].

### 1.2. Limitations of Conventional Analgesic Frameworks

The World Health Organization (WHO) analgesic ladder, introduced in 1986, provides effective relief for approximately 70–80% of patients when applied correctly, but 20–40% of cancer pain is not adequately controlled by the ladder alone and at least 10–15% of patients develop intractable pain requiring advanced interventional approaches [3] (pp. 489–501), [6], [7] (pp. 411–417). Stepwise opioid escalation carries well-documented disadvantages—nausea, constipation, cognitive impairment, respiratory depression, and tolerance—and accumulating preclinical and clinical evidence links perioperative opioid administration to immunosuppression and potentially adverse tumor-related outcomes in oncologic surgery [8] (pp. 106–115).

These limitations have catalyzed interest in what some authors have described as a proposed fourth rung of the analgesic ladder encompassing interventional and neuromodulatory strategies (a framing that is not yet universally adopted), repositioning mechanism-based, anatomically targeted pain management as a first-line consideration rather than a last resort. Despite this growing recognition, access to interventional cancer pain therapies remains substantially limited: a 2026 survey of cancer pain experts reported that permanent PNS and other advanced interventions were consistently limited across practice settings, with the gap between reported utility and actual access representing a major systemic barrier [9] (p. 100727).

### 1.3. Aims of This Review

**Figure 1:**
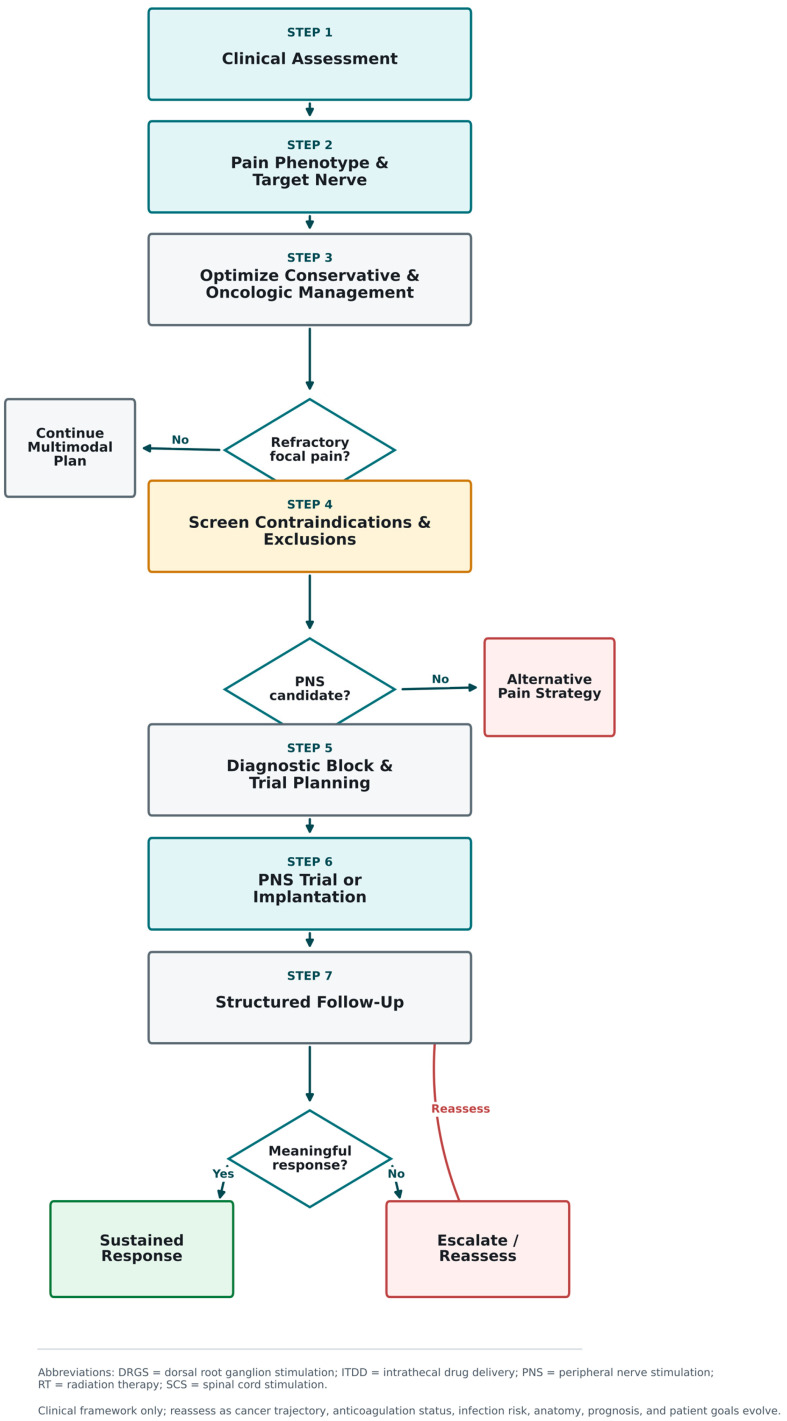
Proposed clinical decision-making algorithm for peripheral nerve stimulation in oncologic and cancer-related pain. The algorithm integrates clinical assessment, pain phenotype and target nerve identification, optimization of conservative and oncologic management, contraindication and exclusion considerations, diagnostic block and trial considerations, PNS candidacy and trial/implantation, and structured follow-up with escalation or reassessment pathways.

Peripheral nerve stimulation—the application of electrical current to a peripheral nerve through a percutaneously implanted electrode—may offer opioid-sparing analgesia, typically without motor blockade, with ambulatory compatibility, reversibility, and technical feasibility under local anesthesia in medically complex patients. These potential advantages must be weighed against practical limitations of the therapy, including the device and procedural cost, the need for ultrasound-guided proceduralist expertise, dependence on a localized, identifiable nerve target, and potential complications such as lead migration or fracture, skin or adhesive reactions, and infection risk in immunocompromised patients. Recent device innovations have enabled widespread adoption of minimally invasive 60-day temporary PNS systems placed in outpatient or procedural settings under ultrasound guidance. Within this clinical context, this narrative review aims to (i) summarize the mechanistic rationale and contemporary device landscape for PNS; (ii) appraise the evidence supporting PNS across oncologic and analogous perioperative pain syndromes; (iii) discuss opioid-sparing and immunological implications relevant to cancer surgery; (iv) propose a pragmatic clinical decision-making framework for PNS candidacy in oncologic populations (see Figure 1); and (v) outline a research agenda to advance the field toward definitive clinical guidance [2] (pp. 118–125), [9], [10] (pp. 691–698), [11] (pp. S115–S191). Although the title emphasizes perioperative care, the “perioperative” window is conceptualized broadly here: in the oncologic setting it encompasses not only acute postoperative analgesia but also the persistent post-surgical and treatment-related pain syndromes (e.g., PTPS, PMPS, plexopathy, and CIPN) that originate from cancer surgery and adjuvant therapy and may extend for months to years beyond the immediate operative episode. This extended scope is intentional and reflects the continuum of cancer-related neuropathic pain to which PNS may be applied.

## 2. Narrative Review Methodology

This article was prepared as a narrative review and is therefore not a systematic review or meta-analysis. To improve methodological transparency, the review process was structured around the six domains of the Scale for the Assessment of Narrative Review Articles (SANRA): (1) justification of the article’s importance, (2) statement of concrete aims or formulation of questions, (3) description of the literature search, (4) referencing and source selection, (5) scientific reasoning and evidence synthesis, and (6) appropriate presentation of relevant endpoint data and clinical applicability. The sections below describe how each domain was addressed. A narrative rather than systematic format was deliberately chosen because the relevant literature spans markedly heterogeneous evidence types—mechanistic and preclinical work, single-arm case series and reports, non-cancer randomized trials, and clinical practice guidelines—that are not amenable to quantitative pooling, and because the review’s aim is to integrate evidence across the mechanistic-to-clinical continuum rather than to answer a single, narrowly defined effectiveness question. Accordingly, the SANRA framework, rather than PRISMA, is the appropriate reporting standard, and the methods below are presented to ensure transparency without overstating the systematic rigor of the search.

### 2.1. Justification and Aims

The justification for this review derives from the high and growing global burden of inadequately controlled cancer pain, the immunological implications of perioperative opioid exposure in oncologic surgery, and the rapid technological evolution of minimally invasive PNS systems, which together create an urgent need for an integrative, clinically oriented synthesis. The specific aims of the review are listed in Section 1.3 and frame the structure of the manuscript.

### 2.2. Literature Search Strategy

A focused literature search was performed from database inception through March 2026 in PubMed/MEDLINE, Embase, and the Cochrane Central Register of Controlled Trials (CENTRAL). Search terms combined neuromodulation concepts (“peripheral nerve stimulation”, “percutaneous peripheral nerve stimulation”, “temporary peripheral nerve stimulation”, “PNS”, “percutaneous electrical nerve stimulation”, “PENS”, “neuromodulation”, “neurostimulation”) with pain-domain terms (“cancer pain”, “oncologic pain”, “neuropathic pain”, “perioperative analgesia”, “post-surgical pain”, “chronic post-surgical pain”), cancer-specific syndromes (“post-mastectomy pain”, “post-thoracotomy pain”, “chemotherapy-induced peripheral neuropathy/CIPN”, “brachial plexopathy”, “lumbosacral plexopathy”), and clinical context terms (“oncologic/cancer surgery”, “ERAS”, “opioid-sparing”, “opioid-free anesthesia”, “perioperative immunosuppression”), connected with Boolean operators. Reference lists of pivotal articles, recent systematic reviews, and clinical practice guidelines were hand-searched to identify additional relevant publications. The initial search and reference-list review were conducted by two authors (T.J. and S.A.), with title and abstract relevance screening followed by full-text review of candidate articles; disagreements regarding inclusion were resolved by discussion and consensus. Reflecting the narrative design, citation selection was guided by author judgment of relevance and quality rather than by a pre-specified protocol, and a formal count of every record screened and excluded was not maintained. The synthesis ultimately draws on approximately 60 primary sources, of which the studies most directly informing clinical and mechanistic conclusions are summarized in Table 1. Within this evidence base, two studies are oncologic-specific clinical reports (one pilot study, n = 12; one retrospective review, n = 15), with the remainder comprising non-cancer randomized trials and systematic reviews, chronic-pain and real-world cohorts, mechanistic and preclinical investigations, and clinical practice guidelines.

Because this is a narrative synthesis without quantitative pooling, no formal risk-of-bias instrument (e.g., RoB 2 or ROBINS-I) was applied to individual studies. Risk of bias is instead addressed qualitatively through the OCEBM evidence grading described in Section 2.4, which signals where conclusions rest on uncontrolled, single-center, or preclinical data. The reader should note that the oncologic-specific evidence base is small and dominated by uncontrolled designs, which carry substantial risk of selection and reporting bias; the potential for publication bias toward positive case reports and small series is also acknowledged, and inferences drawn from extrapolated non-cancer data are identified as such throughout the manuscript.

### 2.3. Referencing and Source Selection

Sources were selected on the basis of their relevance, quality, and contribution to the review’s aims rather than through a pre-specified protocol. Preference was given to peer-reviewed, English-language publications, including randomized controlled trials, prospective and retrospective observational studies, case series and reports for novel or rare indications, systematic reviews and meta-analyses, evidence-based clinical practice guidelines, and mechanistic preclinical investigations with clear translational relevance. Studies focused exclusively on transcutaneous electrical nerve stimulation (TENS) without relevance to percutaneous or implanted PNS systems, as well as studies of central neuromodulation in isolation, were de-emphasized. All cited sources are referenced in the bibliography to support traceability.

### 2.4. Scientific Reasoning and Evidence Synthesis

Evidence was integrated narratively across the mechanistic, clinical-trial, real-world, and guideline domains. Each cited clinical study was assigned a level of evidence using the Oxford Centre for Evidence-Based Medicine (OCEBM) 2011 framework (Level 1, systematic reviews/meta-analyses of RCTs and evidence-based guidelines; Level 2, individual RCTs and prospective comparative studies; Level 3, non-randomized cohort and retrospective comparative studies; Level 4, case-controlled studies and uncontrolled observational case series; Level 5, mechanistic reasoning, expert opinion, and individual case reports). This grading is referenced throughout the manuscript and supports the transparent calibration of confidence in the conclusions drawn. Where directly comparable cancer-specific data were lacking, evidence from analogous non-cancer surgical or chronic pain populations is explicitly identified as extrapolated rather than confirmatory. OCEBM grading is applied selectively to the most directly relevant clinical evidence rather than uniformly to every cited source; mechanistic, background, and supporting references are therefore not all individually labeled.

### 2.5. Presentation of Endpoints and Clinical Applicability

Where available, quantitative analgesic endpoints (e.g., NRS reductions, ≥50% pain-relief responder rates, opioid consumption in morphine milligram equivalents, and validated CIPN and quality-of-life instruments), safety outcomes, and functional measures are reported in the original units provided by primary studies. Clinical applicability is emphasized through a proposed perioperative deployment pathway integrating PNS into ERAS protocols, candidacy criteria distilled from contemporary guidelines, and a clinical decision-making algorithm (Figure 1) that consolidates the assessment, optimization, candidacy, and follow-up steps relevant to oncologic surgical patients.

## 3. Technological Evolution and Mechanism of Action

### 3.1. Historical Context and Device Development

The conceptual foundation of PNS dates to the 1960s with Melzack and Wall’s gate control theory, which proposed that activation of large-diameter non-nociceptive A-beta fibers could suppress nociceptive signal transmission at the spinal dorsal horn—providing the first mechanistic rationale for using electrical stimulation of peripheral nerves to modulate pain perception. Early PNS systems required open surgical implantation with considerable procedural morbidity. Contemporary systems have been transformed through miniaturization, materials innovation, and ultrasound integration. Minimally invasive percutaneous lead platforms, exemplified by the SPRINT PNS System (SPR Therapeutics, Cleveland, OH, USA), use fine-wire, open-coil electrode leads deployable in outpatient procedural settings under local anesthesia and real-time ultrasound guidance [2] (pp. 118–125), [10] (pp. 691–698), [11] (pp. S115–S191), [27] (p. 4540).

The FDA initially cleared the SPRINT system for extremity and back pain; an expanded indication granted in October 2021 extended clearance to the head, neck, and anterior torso, based on real-world safety data from over 5500 treated patients—enabling PNS application to the truncal pain syndromes most prevalent in oncologic surgery. As of March 2026, Aetna’s national coverage designation of SPRINT as medically necessary for intractable neuropathic pain (an estimated 22.3 million covered lives) represents a structural development that may facilitate broader access, although its clinical and population-level impact has not yet been studied and payer policy may change. Parallel device development has yielded additional platforms including the StimRouter (Bioventus, Durham, NC, USA) for chronic peripheral nerve pain, various multi-contact extraforaminal lead systems, and emerging non-implantable magnetic peripheral nerve stimulation devices [28] (pp. S6–S12), [18] (pp. 637–645), [29] (pp. 209–222).

### 3.2. Peripheral, Spinal, and Supraspinal Analgesic Mechanisms

PNS analgesia is multifactorial. Peripherally, PNS downregulates inflammatory mediators (substance P, CGRP, and prostaglandins) at the site of nerve injury, suppresses ectopic neural discharges from neuromas or demyelinated axons, and reduces the expression of pro-inflammatory cytokines and microglial activation markers. Spinally, gate-control mechanisms operate through A-beta fiber activation of inhibitory interneurons—particularly glycinergic and GABAergic neurons in Rexed laminae II–III of the dorsal horn—pre-synaptically reducing A-delta and C-fiber nociceptive transmission to projection neurons in laminae I and V [27] (p. 4540), [28] (pp. S6–S12).

A mechanistic study in a bone-cancer rat model using direct electrical stimulation of the peripheral nerve at 60 Hz and 0.3 mA, parameters broadly analogous to, but not identical to, those used in clinical PNS—demonstrated that stimulation induces Arc (activity-regulated cytoskeleton-associated protein) expression in the spinal dorsal horn. This reduces AMPA-type glutamate receptor signaling at postsynaptic membranes via the Arc-mediated downregulation of GluA1 (transcriptional and, per prior work, post-synaptic AMPAR trafficking), reducing AMPAR-mediated excitatory neurotransmission (OCEBM Level 5)—identifying a biologically specific pathway through which PNS attenuates bone cancer pain at the synaptic level [25] (pp. 599–609).

Supraspinally, PNS engages descending pain-inhibitory pathways including serotonergic projections from the nucleus raphe magnus, noradrenergic fibers from the locus coeruleus, and GABAergic/glycinergic interneuron networks. Functional neuroimaging shows activation of the anterior cingulate cortex, prefrontal cortex, and periaqueductal gray, with no evidence of habituation over decades of follow-up (OCEBM Level 4). The neuroplastic hypothesis for prolonged post-stimulation analgesia holds that sustained reduction of peripheral nociceptive input over a 60-day treatment period disrupts activity-dependent central sensitization—NMDA-mediated long-term potentiation at dorsal-horn synapses and cortical pain-matrix reorganization—yielding persistent reductions in central pain processing that outlast active stimulation [30] (pp. 501–514). Standard parameters range across 10–100 Hz and a 15–200 µs pulse width, with amplitudes titrated to produce comfortable paresthesias without motor activation. Unlike local-anesthetic peripheral nerve blocks, PNS produces neither motor blockade nor sensory deficit, enabling unrestricted early mobilization [2] (pp. 118–125), [9], [10] (pp. 691–698), [11] (pp. S115–S191), [22] (pp. 37–44), [26] (pp. 3117–3139), [15] (pp. 15–27).

## 4. Cancer-Specific Pain Syndromes: Targets and Applications

### 4.1. Post-Thoracotomy Pain Syndrome

Post-thoracotomy pain syndrome (PTPS), pain persisting along a thoracotomy scar for more than two months, occurs in approximately 47% of patients at 6 months after thoracotomy for lung cancer resection and arises from intercostal nerve injury, neurotomy, or periosteal damage, with subsequent central sensitization. Even with pre-emptive thoracic epidural placement, PTPS incidence at six months remains 14.9% versus 42.7% with PCA alone, suggesting that inhibiting central sensitization beyond the acute postoperative period requires longer-duration interventions such as PNS [31] (pp. 887–897), [32].

Percutaneous PNS targeting, affecting thoracic spinal nerve levels under ultrasound guidance, offers a mechanistically appropriate approach to PTPS, with extraforaminal lead placement confirmed by real-time stimulation testing of paresthesia coverage. Oncologic series have included PTPS among cancer pain presentations successfully managed with PNS, with analgesic effects extending significantly beyond the 60-day lead-removal point in responders (OCEBM Level 4) [2] (pp. 118–125), [33], [34] (pp. S38–S40), [20] (pp. 819–826).

### 4.2. Post-Mastectomy Pain Syndrome

Post-mastectomy pain syndrome (PMPS) affects 25–60% of patients after breast cancer surgery and results from injury to the intercostobrachial nerve, pectoral nerves, long thoracic nerve, and thoracodorsal nerve. With over 2.3 million new breast cancer diagnoses annually, the population-level burden is substantial. Despite gabapentinoids, tricyclic antidepressants, SNRIs, pulsed radiofrequency, and regional blocks, no treatment is universally effective [35] (pp. 725–746), [36] (pp. 1807–1815), [37].

PNS is increasingly recognized in PMPS algorithms as a viable option for refractory cases. Percutaneous targeting of specific intercostal or thoracic spinal nerve roots without motor block or sensory deficit is particularly advantageous where ipsilateral upper-extremity function and rehabilitation (including lymphedema management) are critical priorities. ASRA guidelines acknowledge PNS among interventional options for PMPS management, and oncologic PNS series demonstrate efficacy in post-mastectomy presentations [33,37], [38] (pp. 44–51).

### 4.3. Brachial and Lumbosacral Plexopathies

Tumor invasion or radiation-induced injury to the brachial or lumbosacral plexus produces some of the most refractory pain presentations in oncology. Ultrasound-guided percutaneous PNS of the inferior trunk of the brachial plexus—targeting the C8/T1 convergence supraclavicularly with the subclavian vein as a vascular landmark—has been reported in cancer patients with plexopathy-related upper-extremity neuropathic pain (OCEBM Level 5). The supraclavicular approach enables lead placement proximal to the lesion. These cases underscore the importance of individualized pre-procedural cross-sectional imaging (MRI or CT) for anatomy mapping where tumor invasion or prior surgical/radiation-related distortion may alter normal nerve topography [33].

### 4.4. Chemotherapy-Induced Peripheral Neuropathy

Chemotherapy-induced peripheral neuropathy (CIPN) affects 30–40% of patients receiving neurotoxic agents (platinum, taxanes, vinca alkaloids, proteasome inhibitors, and immunomodulatory agents). Approximately 30–40% develop chronic symptoms persisting beyond chemotherapy, and >50% of taxane-treated patients report persistent neuropathy at one year. Despite NCI sponsorship of 15 pharmacological trials, only duloxetine has demonstrated benefit in a single RCT (OCEBM Level 1). CIPN pathophysiology involves dorsal-root-ganglia neuronal apoptosis, axonal-transport disruption, mitochondrial dysfunction, and oxidative stress, producing large-fiber and small-fiber phenotypes [39] (pp. 772–781), [40] (pp. 3325–3348).

A 2025 case report described a 75-year-old woman with colorectal adenocarcinoma and oxaliplatin-induced CIPN treated with a 12-week course of percutaneous electrical nerve stimulation (PENS) targeting the median nerve (1 Hz, 200 µs, 1.3 mA, 30 min/session) without chemotherapy interruption (OCEBM Level 5). Progressive improvement was documented with EORTC QLQ-CIPN20 and quantitative sensory testing across all somatosensory fiber classes, with no device-related adverse events. A 2023 Johns Hopkins preclinical study demonstrated that spinal cord stimulation reduced neuropathic pain behaviors in rats implanted with human lung cancer tissue receiving paclitaxel without compromising—and in fact enhancing—antitumor efficacy. A retrospective series further showed successful treatment of lower-extremity CIPN with dorsal root ganglion stimulation (DRGS), particularly for distal, well-localized symptoms (OCEBM Level 4) [40] (pp. 3325–3348), [24] (p. 133), [41] (pp. 938–949).

It should be emphasized that the evidence for PNS specifically in CIPN is at present the most preliminary of the syndromes discussed here—comprising a single case report, one preclinical spinal cord stimulation study, and a retrospective DRGS series rather than direct PNS trial data—and is therefore appreciably weaker than the evidence supporting PNS for focal post-surgical syndromes such as PTPS and PMPS. There is also a mechanistic caveat: CIPN pathophysiology is driven substantially by dorsal-root-ganglia neuronal apoptosis, axonal-transport disruption, and mitochondrial injury, processes that are not obviously addressed by the peripheral A-beta-mediated gate-control mechanism underpinning PNS’s efficacy in focal neuropathic pain. PNS may nonetheless modulate the symptomatic, ectopic-discharge component of CIPN, but its role should be regarded as investigational, and the relative prominence of this section reflects the clinical importance of the unmet need rather than the maturity of the supporting evidence.

### 4.5. Truncal and Abdominal Neuropathic Pain

Neuropathic abdominal/truncal pain is highly prevalent in gastrointestinal, gynecological, and genitourinary malignancies and after major abdominal cancer surgery. A 2024 technical report described a medial-to-lateral ultrasound-guided thoracoabdominal nerve PNS technique via a transversus abdominis plane (TAP) approach, using an eight-contact non-tined lead between the internal oblique and transversus abdominis to simultaneously cover the ilioinguinal, iliohypogastric, and lateral femoral cutaneous nerves (OCEBM Level 5). This extends the anatomical repertoire of PNS to abdominal neuropathic pain syndromes that previously lacked a reliable percutaneous neurostimulation target [42] (pp. 981–987).

## 5. Evidence-Graded Review of Key Clinical Studies

### 5.1. Evidence Summary Table

Table 1 summarizes the primary studies informing evidence on PNS for perioperative and oncologic pain management, categorized by their OCEBM evidence level. To prevent the conflation of evidence with differing external validity, the studies are grouped by their population relevance to oncology: (i) oncologic-specific clinical evidence directly applicable to cancer patients (Mainkar et al. 2020 [20]; the 2024 oncologic retrospective; the CIPN PENS case report; and the bone-cancer preclinical model); (ii) perioperative non-oncologic evidence considered analogous to the cancer-surgery setting (Ilfeld et al. trial [43] (PAINfRE investigators), the TKA RCT, the post-amputation RCT, Gabriel et al. [19], and the orthopedic systematic reviews); (iii) chronic and neuropathic pain evidence and real-world/long-term cohorts; and (iv) mechanistic and guideline evidence. Readers should note two interpretive caveats. First, the reported analgesic endpoints are based on heterogeneous definitions of “success” (e.g., ≥50% pain relief, patient global impression of change, or absolute NRS thresholds) and varying follow-up durations and sample sizes, so the oncologic success rates of 58–67% and the responder rates cited from non-cancer cohorts are not directly comparable and should not be pooled. Second, where adverse-event rates and primary-endpoint definitions are reported in the source studies they are described in the relevant text (Section 5.2, Section 5.3, Section 5.4 and Section 8) rather than tabulated, given the heterogeneity of reporting across designs.

### 5.2. Landmark Randomized Controlled Trials (OCEBM Level 2)

The Ilfeld et al. [17] (pp. 847–861) trial in association with the PAINfRE investigators: This multicenter, randomized, double-blind, sham-controlled trial of percutaneous PNS after ambulatory orthopedic surgery found that the median opioid consumption over postoperative days 1–7 was 5 mg MME (PNS) versus 48 mg (sham) (geometric-mean ratio 0.20; 97.5% CI 0.07–0.57; *p* < 0.001), with an average pain intensity of 1.1 ± 1.1 versus 3.1 ± 1.7 (difference −1.8; 97.5% CI −2.6, −0.9; *p* < 0.001). For brachial plexus leads, active stimulation produced NRS 0.8 [IQR 0.5–1.6] versus 3.2 [2.7–3.5] (*p* < 0.001), with an opioid consumption of 10 mg (5–20) versus 71 mg (35–125) (*p* = 0.043).

Total Knee Arthroplasty RCT (Goree, Grant et al., Neuromodulation, 2024 [17] (pp. 847–861)):The 60-day percutaneous PNS for persistent postoperative pain after TKA met its primary endpoint, with 60% of active-stimulation subjects achieving ≥50% pain relief during weeks 5–8 versus 24% in sham (*p* = 0.028). It also produced a 54% versus 26% mean reduction in pain scores (*p* < 0.05) and +47% improvement in 6 min walk-test distance versus −9% in controls. PGIC ratings improved in 90% of PNS subjects versus 55% of sham (*p* < 0.05). This trial is directly applicable to oncologic populations, as persistent postoperative pain after TKA develops via central-sensitization mechanisms analogous to chronic post-surgical pain following major cancer resections.

Post-Amputation Pain RCT (Gilmore et al., RAPM, 2019) [18] (pp. 637–645): A total of 58% of PNS subjects achieved ≥50% pain relief during weeks 1–4 versus 14% placebo (*p* = 0.037); at 8 weeks, this was 67% versus 14% (*p* = 0.014), with 80% achieving ≥50% reduction in pain interference (*p* = 0.003). Among baseline opioid users, 50% (PNS) versus 43% (placebo) reduced or discontinued analgesics. The direct relevance applies to oncologic limb-amputation patients with sarcoma, melanoma, or other musculoskeletal malignancies.

Lower-Limb PNS Meta-Analysis (Lin et al., 2025 [15] (pp. 15–27)): Eight RCTs (n = 633) showed a marginally significant reduction in analgesic consumption without significant differences in pain intensity, range of motion, or length of stay on pooled analysis (OCEBM Level 1). The authors attributed inconclusive findings to clinical heterogeneity, small samples, and variable stimulation parameters—a counterbalance underscoring the need for methodological rigor in future oncologic PNS studies.

### 5.3. Oncologic-Specific Evidence (OCEBM Level 4)

Mainkar et al. (Neuromodulation, 2020) [20] (pp. 819–826): The landmark pilot study of PNS in oncologic pain enrolled 12 patients and achieved success in 7 (58.3%) across PTPS, PMPS, and post-herpetic neuralgia in immunocompromised cancer patients. Several subjects experienced prolonged analgesia after lead extraction, consistent with the neuroplastic hypothesis. The study established proof of concept for PNS across multiple oncologic pain phenotypes and identified the brachial plexus and spinal nerve targets as clinically viable (OCEBM Level 4).

2024 Oncology Retrospective Review (Neuromodulation): Fifteen oncologic patients were classified into tumor-related, treatment-related, cancer-associated, and cancer-independent pain subtypes; 10/15 (67%) were successful, with treatment-related pain experiencing the least benefit—an actionable patient-selection insight given that treatment-related pain may involve more diffuse or centrally predominant mechanisms (OCEBM Level 4) [2] (pp. 118–125).

Vu et al. Scoping Review (Neuromodulation, 2025) [12] (pp. 191–203): A scoping review (OCEBM Level 4) screened 831 references and included 24 studies (16 SCS, 7 PNS, and 2 DRGS—authors report 24). PNS-specific analysis reported NRS decreases from a baseline mean of 8.29 to 3.04 over an average 5.2-month follow-up, with several patients experiencing prolonged analgesia after lead extraction. Evidence was graded as limited overall owing to the absence of prospective comparative studies, heterogeneity, and small samples—findings consistent with the limitations identified in this review.

### 5.4. Real-World and Long-Term Durability Evidence (OCEBM Level 4)

A cross-sectional real-world survey of 252 patients ≥3 months post-60-day PNS reported a 73% end-of-treatment success rate, with 61% of initial responders maintaining benefits at 3–30 months (58% at ≥12 months). A separate long-term outcomes study at 2–3 years reported a 36.7% long-term response rate, with long-term responders demonstrating PROMIS Pain Intensity score improvements of −9.0 versus +3.1 in non-responders (*p* < 0.0001). The 24-month outcomes data report an 85% responder rate and 67% pain reduction versus baseline, with an 82% responder rate at 18 months; this 24-month durability evidence derives from the COMFORT randomized controlled trial of a permanently implanted micro-implantable pulse generator (micro-IPG) system rather than a temporary 60-day percutaneous PNS system, and the trial excluded patients with active malignancy. A 2025 RAPM systematic review/meta-analysis pooled evidence of sustained analgesia up to 24 months across temporary and permanent PNS systems (OCEBM Level 1). A systematic review of temporary PNS for orthopedic surgery (Harris et al., 2025) identified nine studies (six RCTs, three case series), concluding that tPNS is safe and may reduce postoperative pain and opioid use (OCEBM Level 1) [22] (pp. 37–44), [13,14], [21] (pp. 611–621), [23] (p. 179).

## 6. Acute Postoperative Pain Management in Oncologic Surgery

Ultrasound-guided percutaneous PNS has demonstrated efficacy for acute postoperative analgesia across diverse procedures. A secondary analysis of the Ilfeld et al. [17] (pp. 847–861) trial (PAINfRE investigators) found that for sciatic-nerve leads, active PNS produced NRS 0.7 [0–1.4] versus 2.8 [1.6–4.6] in sham (*p* < 0.001), with an opioid consumption of 5 mg (0–30) versus 40 mg (20–105) (*p* = 0.004) [43] (pp. 638–649). A retrospective analysis of 185 patients corroborated these findings with a 16.8% reduction in 60-day postoperative pain (*p* < 0.001). For ERAS integration in lung cancer surgery specifically, a randomized trial demonstrated lower postoperative pain (2.14 ± 0.86 vs. 3.78 ± 1.15; *p* = 0.017) and improved pulmonary function, illustrating the clinical value of multimodal approaches in thoracic oncology [16] (pp. 95–110), [44], [45] (pp. 342–348).

Several features distinguish PNS in this context. Leads can be optimally placed 2–7 days postoperatively—targeting the transitional phase between acute inflammatory pain and central sensitization. The 60-day treatment duration substantially outlasts continuous peripheral-nerve catheters (typically 3–5 days). The absence of motor blockade enables unrestricted weight-bearing, respiratory physiotherapy, and rehabilitation from the day of lead placement—particularly relevant in thoracic oncology where diaphragmatic splinting and impaired cough mechanics secondary to phrenic nerve involvement from paravertebral or intercostal anesthesia contribute to pulmonary complications [2] (pp. 118–125), [9,27], [35] (pp. 725–746), [17] (pp. 847–861).

## 7. Opioid-Sparing Benefits and Oncologic Immunological Implications

### 7.1. The Perioperative Immunological Window

The perioperative period following cancer surgery represents a critical immunological window during which the capacity to eliminate circulating tumor cells shed during surgical manipulation is compromised. Three converging mechanisms contribute: (1) surgical trauma activates the HPA axis, releasing immunosuppressive glucocorticoids and catecholamines that impair lymphocyte proliferation and natural killer (NK) cell function; (2) volatile halogenated anesthetics suppress NK cell cytotoxicity, T-cell proliferation, and macrophage activity; and (3) opioid analgesics compound immunosuppression via multiple receptor-mediated mechanisms. Strategies that attenuate any of these components—regional anesthesia, total intravenous anesthesia with propofol, and opioid minimization through neuromodulation—may improve the perioperative immune microenvironment and reduce the risk of metastatic seeding [8] (pp. 106–115), [45] (pp. 342–348), [46] (pp. 35–58).

### 7.2. Opioid-Specific Immunological Effects

Fentanyl, sufentanil, and alfentanil each reduce NK cell cytotoxic activity; remifentanil completely inhibits lymphocyte proliferation and NK cell activity at clinically relevant concentrations. Morphine and other opioids impair macrophage phagocytic activity, reduce neutrophil chemotaxis and superoxide production, diminish dendritic-cell antigen presentation, and attenuate cytokine-mediated immune cell recruitment. Beyond immunosuppression, mu-opioid receptor (MOR) activation on vascular endothelial cells promotes angiogenesis via VEGF upregulation and stimulates tumor cell proliferation via MOR expressed on tumor cell surfaces. Methylnaltrexone, a peripheral MOR antagonist, reduces opioid-induced angiogenesis in preclinical models, supporting the rationale for peripheral opioid avoidance in cancer surgery [8] (pp. 106–115), [45] (pp. 342–348).

### 7.3. Clinical Evidence for Opioid-Sparing Impact

A prospective randomized study showed that opioid-free anesthesia preserved M1 macrophage polarization in gastric cancer patients undergoing gastrectomy, while opioid-based anesthesia shifted polarity toward the immunosuppressive M2 phenotype (OCEBM Level 2). A large multicenter RCT reported no significant difference in breast cancer recurrence between regional versus general anesthesia/opioids; while neutral, this does not refute the broader immunological hypothesis. An opioid-sparing multimodal protocol for lumpectomy produced superior pain control compared to opioid-based management without the immunological costs of systemic opioid exposure (OCEBM Level 2). Although a direct causal link between PNS-mediated opioid reduction and improved cancer survival has not been established prospectively, mechanistic data—from NK cell function to macrophage polarization to MOR-mediated tumor angiogenesis—provide compelling rationale for prioritizing opioid minimization, including via PNS, in oncologic surgery [8] (pp. 106–115), [37], [45] (pp. 342–348), [46] (pp. 35–58).

These mechanistic data should, however, be interpreted against the perioperative oncology literature that remains genuinely controversial and, to date, inconsistent. The most prominent counterpoint is the large multicenter randomized controlled trial by Sessler et al. (Lancet, 2019) [36] (pp. 1807-1815), which found no significant difference in breast cancer recurrence between paravertebral–propofol regional anesthesia and sevoflurane–opioid general anesthesia (OCEBM Level 2). This neutral result directly challenges the clinical translation of the opioid–immunosuppression hypothesis and indicates that reductions in opioid-related natural killer cell suppression or MOR-mediated tumor signaling demonstrated in preclinical and in vitro work have not been shown to alter clinically meaningful oncologic outcomes. Translational findings across the broader anesthesia–oncology literature are similarly heterogeneous, in part because it is difficult to disentangle the immunologic effects of opioids from those of surgical stress, the anesthetic technique as a whole, and the underlying tumor biology. Readers should therefore not infer that the opioid–cancer relationship is settled: opioid-sparing is biologically plausible and worth pursuing, but a reduction in perioperative opioid exposure has not been demonstrated to improve cancer recurrence or survival, and the rationale for opioid minimization via PNS in cancer surgery should be regarded as hypothesis-generating rather than as evidence of an oncologic outcome benefit.

## 8. Safety and Adverse Event Profile

Across clinical trials, real-world series, and the accumulated experience underlying the FDA’s 2021 expanded indication (5500+ patients), the safety profile of percutaneous PNS has been consistently favorable. In PAINfRE, device-related events included one pulse-generator malfunction (replaced), one withdrawal for unpleasant sciatic-nerve sensations, and two lead fractures (3%) found on removal without clinical consequences (OCEBM Level 2). In the post-amputation RCT, 22 study-related events occurred in 46% of subjects (n = 13/28), all insignificant—skin irritation/redness, adhesive reactions, mild implantation- or stimulation-related pain, pruritus, and fatigue. No leads fractured during treatment; 5/34 (15%) fractured at the distal tip during intentional removal without clinical sequelae. Across major trials, no serious unanticipated device-related events, permanent nerve injuries, or infections requiring antibiotic therapy have been reported [29] (pp. 209–222), [38] (pp. 44–51), [16] (pp. 95–110), [17] (pp. 847–861), [47] (pp. 170–180).

Oncologic-specific risk considerations include lead-placement proximity to tumor beds or post-radiation tissue, immunocompromise during active systemic therapy potentially increasing infection susceptibility, and interaction with concurrent radiation fields. Absolute contraindications include active local infection at the proposed electrode site, severe coagulopathy precluding percutaneous needle insertion, and inability to cooperate with paresthesia confirmation. Anticoagulants and antiplatelet agents require individualized assessment following ASRA guidelines. Placement under local anesthesia without sedation allows PNS deployment in patients with significant cardiopulmonary, hepatic, or other comorbidities that might otherwise preclude procedural interventions—a particular advantage in cancer patients [9], [10] (pp. 691–698), [11] (pp. S115–S191).

## 9. Technical Considerations and Lead Placement

### 9.1. Contemporary Device Technology

Contemporary PNS systems use fine-wire, open-coil electrode leads (~0.2 mm diameter) inserted through a coaxial-gauge needle under real-time ultrasound guidance, with the electrode advanced beyond the needle tip to rest adjacent to the target nerve. Open-coil architecture enables flexibility, reducing fracture rates, while the small gauge minimizes tissue trauma and infection risk. An external pulse generator (EPG) delivers programmable biphasic or monophasic waveforms via a semi-implanted connector. The 60-day dwell time supports tissue integration and mechanical stabilization while maintaining reversibility and avoiding the permanent implant surgery required for traditional pulse-generator placement [2] (pp. 118–125), [9], [11] (pp. S115–S191), [37], [45] (pp. 342–348).

### 9.2. Ultrasound-Guided Placement Techniques

Ultrasound enables the real-time visualization of target neural structures, vasculature, and tissue planes. For thoracic spinal nerve targets relevant to PTPS and PMPS, a prone extraforaminal approach uses a high-frequency (12–15 MHz) linear transducer; the needle is introduced in-plane laterally to medially with stimulation testing confirming paresthesia coverage. For peripheral targets, lower-frequency ultrasound (8–12 MHz) visualizes nerve fascicle anatomy for sub-epineural placement. For truncal abdominal pain via TAP, the lead is placed parallel to the internal-oblique/transversus-abdominis fascial plane for multi-nerve coverage. Magnetic peripheral nerve stimulation represents an emerging non-implantable platform with potential advantages in the most medically fragile patients, supported by preliminary safety and feasibility data [2] (pp. 118–125), [10] (pp. 691–698), [11] (pp. S115–S191), [18] (pp. 637–645), [42] (pp. 981–987).

## 10. Patient Selection and Clinical Indications

### 10.1. Evidence-Based Candidacy Criteria

The 2024 ASIPP Comprehensive Evidence-Based Guidelines established Level III evidence (moderate certainty) for temporary 60-day PNS in moderate-to-severe chronic pain refractory to conservative treatment (OCEBM Level 1). The 2025 ASPN Consensus Guidelines provide criteria for PNS candidacy covering neuropathic, post-surgical, and chronic pain indications, including stimulation parameters, lead placement, patient selection, and complication management (OCEBM Level 1) [11] (pp. S115–S191), [26] (pp. 3117–3139).

For oncologic patients, optimal PNS candidates share several characteristics [8] (pp. 106–115), [40] (pp. 3325–3348), [46] (pp. 35–58):

- Pain localized to one or several identifiable peripheral nerve distributions or dermatomal territories.
- Predominantly neuropathic character (burning, shooting, allodynia, or dysesthesia) or mixed nociceptive/neuropathic presentation.
- Moderate-to-severe pain intensity (NRS ≥ 4) inadequately controlled by optimized pharmacological management.
- Intermediate survival prognosis (weeks to months) sufficient to benefit from a 60-day treatment course.
- Absence of absolute contraindications to percutaneous lead placement.
- Adequate coagulation status and absence of active infection at the planned electrode site.
- Medically stable enough to tolerate an outpatient procedural intervention under local anesthesia.
- Motivation and cognitive capacity to operate the EPG and provide symptom feedback.

### 10.2. Pain Subtype Differential Response

The 2024 oncologic retrospective demonstrated differential outcomes across cancer pain subtypes: tumor-related, cancer-associated, and cancer-independent pain achieved comparable success, whereas treatment-related pain showed the least response—likely reflecting more diffuse or centrally predominant mechanisms. Patients with diffuse or rapidly evolving pain from widespread metastatic disease are generally better served by intrathecal drug delivery or systemic strategies, as PNS requires a localized target [2] (pp. 118–125), [48], [49] (p. 117).

### 10.3. Psychological Screening and Optimization

Pre-PNS depression significantly predicts non-response, with long-term responders showing lower depressive symptoms at baseline (PROMIS Depression T-score 50.3 ± 10.7 vs. 57.9 ± 8.9; *p* = 0.05) (OCEBM Level 4). Depression and anxiety affect 30–40% of cancer patients and are frequently undertreated. Routine pre-procedural assessment using validated instruments (PHQ-9 and PROMIS Depression) enables identification of patients requiring psychological optimization prior to device implantation; a multidisciplinary approach incorporating psychology, social work, and palliative care is likely to improve patient selection and outcomes [8] (pp. 106–115), [22] (pp. 37–44), [26] (pp. 3117–3139).

### 10.4. Proposed Clinical Decision-Making Algorithm

A pragmatic clinical decision-making algorithm consolidating the candidacy, optimization, contraindication, trial, and follow-up considerations described above is provided as Figure 1.

## 11. PNS Within the Broader Neuromodulation Landscape

### 11.1. Comparison with Spinal Cord Stimulation

Spinal cord stimulation (SCS) has a longer evidence history in cancer pain, with NRS reductions from 8.0 to 2.2 over 8.4-month follow-up in 16 oncologic studies included in the Vu et al. [12] (pp. 981–987). scoping review. A comparative retrospective study demonstrated that patients with neuropathic pain of peripheral nerve origin achieved higher rates of positive SCS trials (OR = 9.60; 95% CI 1.48–62.16; *p* = 0.018) and superior long-term NPRS reductions versus central-lesion origin pain. For cancer patients, PNS offers practical advantages over SCS: placement under local anesthesia without sedation; avoidance of epidural space access (critical in spinal metastases or coagulopathy); reversibility; and lower upfront procedural complexity. SCS is generally preferred for more diffuse, multidermatomal, or lower-extremity-predominant patterns [2] (pp. 118–125), [9], [12] (pp. 191–203), [48] (p. 102124), [49] (p. 117).

### 11.2. Comparison with Dorsal Root Ganglion Stimulation

DRGS targets the sensory ganglion at the spinal foramen, offering focused dermatomal coverage without postural variability. DRGS has shown promising results for lower-extremity CIPN, with a retrospective series reporting successful long-term implantation for isolated foot/toe CIPN refractory to SCS (OCEBM Level 4). PNS is generally preferred where pain is localized to a specific peripheral nerve accessible to ultrasound-guided lead placement, where placement must occur under local anesthesia without fluoroscopy, or where a temporary, reversible approach is desired. DRGS requires fluoroscopy, transforaminal epidural access, and permanent implantation [41] (pp. 938–949), [47] (pp. 170–180).

### 11.3. Comparison with Intrathecal Drug Delivery

Intrathecal drug delivery (ITDD)—morphine, hydromorphone, ziconotide, or combinations—remains the reference standard for diffuse, severe, refractory cancer pain. PNS is not an appropriate substitute for ITDD in diffuse cancer pain or when systemic opioid requirements are so high that pump-delivered analgesia is required. Rather, PNS and ITDD occupy complementary niches; combination therapy (ITDD for background analgesia plus PNS for focal neuropathic overlay) may represent an optimal approach in selected patients with mixed pain presentations, though formal study is lacking [50] (pp. 355–367).

## 12. Integration into Multimodal Perioperative Pathways

Contemporary perioperative care emphasizes multimodal, opioid-minimizing strategies as foundational elements of ERAS protocols for cancer surgery. ERAS in elderly NSCLC patients undergoing lung resection produced lower postoperative pain (2.14 ± 0.86 vs. 3.78 ± 1.15; *p* = 0.017), reduced pulmonary complications, and improved FVC and FEV1 on postoperative day 7. In colorectal cancer surgery, some studies have associated ERAS compliance with survival improvements, though results are inconsistent, suggesting that the cumulative physiological benefits of opioid reduction, early mobilization, and accelerated recovery may extend beyond the perioperative episode [16] (pp. 95–110), [17] (pp. 847–861).

PNS integrates naturally into ERAS frameworks. A strategic perioperative PNS deployment protocol for major cancer surgery includes several phases [27,37], [51] (pp. E131–E152), [52] (pp. 1–131): this six-phase protocol should be understood as a consensus, expert-opinion framework (OCEBM Level 5) intended to organize the clinical workflow rather than as an evidence-derived pathway; in particular, the recommended window for lead placement (postoperative days 2–7) reflects the mechanistic rationale of targeting the transition from acute inflammatory pain to central sensitization, and has not been established by prospective timing studies.

- Preoperative phase: This should involve candidacy screening (pain localization, psychological assessment, coagulation, and anatomical feasibility); incorporation of PNS into ERAS consent and counseling; and consideration of preoperative PNS in patients with pre-existing cancer pain.
- Intraoperative phase: This should involve an optimized regional anesthetic technique (paravertebral, serratus anterior, or TAP block as appropriate); an opioid-sparing or opioid-free anesthetic strategy; and avoidance of volatile agents where feasible.
- Acute postoperative phase (days 0–2): This should involve multimodal pharmacological analgesia (acetaminophen, NSAIDs when appropriate, and gabapentinoids); the regional technique wearing off; and pain assessment confirming PNS eligibility.
- PNS placement phase (days 2–7): This should involve ultrasound-guided percutaneous lead placement targeting the operative field’s peripheral nerve distribution; stimulation testing and securing; and ERAS continuation of early mobilization and oral intake.
- Extended recovery phase (days 7–60): This should involve active PNS concurrent with oral multimodal analgesia; progressive opioid tapering; physical therapy without motor blockade; and transition to ambulatory care with portable EPG.
- Post-60-day phase: This should involve lead removal; assessment for sustained post-stimulation analgesia; planning of adjuvant pain management as needed; and the transition to definitive analgesic strategy.

## 13. Economic Considerations and Access

### 13.1. Cost-Effectiveness

Preliminary health-economic analyses suggest that PNS may offer cost-effective alternatives to traditional analgesic strategies once opioid-related adverse events, prolonged stays, ED visits, and readmissions are accounted for. The 2024 Ontario Health Technology Assessment evaluated PNS in chronic neuropathic pain. Temporary 60-day percutaneous systems avoid the upfront device/operative costs of permanent implantable pulse generators while providing sufficient duration for perioperative and subacute recovery phases. Real-world utilization data further support the cost-offset potential [31] (pp. 887–897), [51] (pp. E131–E152), [52] (pp. 1–131), [53], [54] (pp. 27–36), [55].

### 13.2. Reimbursement Landscape

The 2021 FDA expanded clearance extended the SPRINT system’s indication to the head, neck, and anterior torso, enabling PNS for the truncal syndromes most relevant to oncologic surgery. Aetna’s March 2026 national coverage designation (an estimated 22.3 million covered lives) represents a structural shift enabling access across a substantial segment of the commercially insured population. Medicare coverage for temporary percutaneous PNS remains inconsistently defined across carriers. Securing dedicated reimbursement pathways for temporary PNS in oncologic patients will require cancer-specific cost-effectiveness evidence and advocacy through oncology and pain medicine societies [29] (pp. 209–222), [56] (pp. 269–282).

## 14. Evidence Limitations and Knowledge Gaps

Several limitations constrain definitive conclusions. The available cancer-specific literature comprises a single pilot study (n = 12), one retrospective review (n = 15), and seven studies included in the Vu et al. [12] (pp. 981–987). scoping review (OCEBM Level 4), with explicit acknowledgment of an absence of prospective comparative studies, clinical heterogeneity, and small samples. Robust Level 2 RCT evidence supporting PNS in orthopedic and chronic pain populations cannot be assumed to extrapolate directly to oncologic patients, who are distinguished by immunocompromise, tumor-related anatomical distortion, concurrent systemic therapies, and rapidly evolving pain profiles [2] (pp. 118–125), [20] (pp. 819–826), [40] (pp. 3325–3348), [12] (pp. 191–203), [46] (pp. 35–58).

Additional limitations include: the absence of oncologic-specific RCT data for any cancer pain syndrome; reliance on single-center, retrospective, or uncontrolled designs in available oncologic evidence; lack of standardized outcome measures; limited follow-up duration relative to disease trajectories; underrepresentation of patients on active systemic therapy in safety datasets; and absence of comparative effectiveness data versus other interventional cancer pain strategies. The evidence for CIPN, brachial plexopathy, and abdominal cancer pain is limited to case reports and preclinical models. Device-specific factors—electrode design, waveform, and lead positioning—vary across the literature, complicating cross-study comparisons.

### Practical Limitations and Implementation Challenges

Beyond the limitations of the evidence base, several practical limitations of the therapy itself warrant explicit acknowledgment for a balanced, clinically realistic appraisal. PNS requires a localized, identifiable peripheral-nerve target and is therefore unsuited to diffuse, multifocal, or rapidly progressive metastatic pain. Successful deployment depends on ultrasound-guided proceduralist expertise: lead placement, real-time paresthesia mapping, and management of the external pulse generator carry a meaningful learning curve, and operator dependence may limit reproducibility outside specialized centers. Device- and procedure-related considerations include lead migration or fracture (most commonly at intentional removal), skin and adhesive reactions, the need for a cooperative, cognitively intact patient able to operate the device, and the cost of the system and procedure relative to inconsistent reimbursement. Implementation within an oncologic ERAS pathway additionally requires coordinated scheduling around chemotherapy, radiotherapy, and surgery, and access to multidisciplinary support.

Training requirements and operator dependence therefore represent a genuine barrier to scalable adoption, and structured proctoring, credentialing, and procedural-volume thresholds are likely to be important for safe dissemination. Finally, certain oncologic subpopulations may represent unique procedural-risk groups that have been underrepresented in existing safety datasets. Patients receiving immune checkpoint inhibitors or other immunotherapy may have altered inflammatory and wound-healing responses and theoretically heightened infection or immune-related adverse-event risk around a percutaneous indwelling lead, while patients on antiangiogenic agents (e.g., VEGF-pathway inhibitors) carry recognized risks of impaired wound healing and bleeding that may bear on the timing of lead placement and removal. These considerations are currently based on extrapolation rather than dedicated study, and individualized, multidisciplinary risk assessment—including coordination with the treating oncologist regarding the timing of systemic therapy—is advisable until prospective safety data in these populations become available.

## 15. Future Research Directions

### 15.1. Randomized Controlled Trials in Oncologic Populations

Large-scale, multicenter, adequately powered RCTs evaluating PNS in defined oncologic surgical populations—thoracotomy/thoracoscopy for lung cancer, mastectomy/axillary dissection for breast cancer, limb-sparing or amputation surgery for sarcoma, and gastrointestinal cancer resection—are the highest methodological priority. Trials should employ active sham controls, blinded outcome assessment, and pre-specified oncologic endpoints alongside pain measures, including EORTC QLQ-C30, FACT-G, opioid MME, ERAS adherence, and time to functional milestones. Powering should target detection of a minimum 30% opioid reduction as primary endpoint [46] (pp. 35–58), [51] (pp. E131–E152).

### 15.2. Cancer Recurrence and Immunological Outcome Studies

Prospective trials examining the relationship between PNS-mediated opioid reduction and cancer recurrence, disease-free survival, and overall survival are needed. Biomarker substudies measuring NK cell cytotoxic activity, T-cell subsets and clonal diversity, macrophage polarization indices, and cytokine profiles (IL-6, TNF-α, and IL-10) at perioperative timepoints would mechanistically corroborate and extend preclinical findings. Preclinical work using established cancer pain models should evaluate the effects of PNS on tumor growth, metastasis, and chemotherapy efficacy under controlled conditions [8] (pp. 106–115), [45] (pp. 342–348), [57] (pp. 4–6), [58].

### 15.3. CIPN-Specific Trials

Phase II/III trials of percutaneous PNS and PENS for CIPN across major neurotoxic classes (platinum, taxane, vinca alkaloid, and proteasome inhibitor) are a high-value priority, standardizing nerve targets, stimulation parameters, and session protocols; using validated CIPN instruments (EORTC QLQ-CIPN20, and FACT/GOG-Ntx); and assessing whether PNS enables maintenance of full chemotherapy dosing—a directly oncological endpoint [39] (pp. 772–781), [40] (pp. 3325–3348), [24] (p. 133).

### 15.4. Comparative Neuromodulation Effectiveness Research

Head-to-head comparative effectiveness trials of PNS, SCS, DRGS, and ITDD in matched oncologic populations would clarify their relative positionings across pain phenotypes, prognosis categories, functional profiles, and care settings. An adaptive design enabling crossover between modalities based on response would be ethically appropriate and methodologically efficient [3] (pp. 489–501), [48].

### 15.5. Optimal Stimulation Protocol Determination

Comparative trials of frequency (conventional 40–100 Hz vs. low-frequency 1–10 Hz vs. burst/high-density), pulse width, amplitude, duty cycle, and treatment duration are needed to define optimal protocols across phenotypes. Whether the 60-day standard duration is necessary, sufficient, or optimal remains untested, as do adaptive dosing strategies extending the duration for partial responders or shortening it for full responders [9], [26] (pp. 3117–3139).

### 15.6. Psychological Predictors and Optimization Protocols

Prospective validation of psychological predictors of PNS response—depression, anxiety, catastrophizing, and resilience—in oncologic populations will enable refined selection algorithms. Brief pre-procedural psychological optimization (e.g., single-session ACT for pain and problem-solving therapy for cancer-related depression) may improve outcomes in patients with a baseline depressive burden [22] (pp. 37–44).

### 15.7. Health Economic Analyses and Implementation Science

Implementation-science research examining the workflow, institutional requirements, and coordination pathways for PNS within cancer-specific ERAS protocols would provide actionable guidance for adoption. Embedded health-economic analyses should incorporate cancer-specific downstream costs—opioid-related adverse events, oncologic readmissions, and potentially long-term implications of recurrence—to generate comprehensive models for payer and health-system decision-making [50], [54] (pp. 27–36), [55] (pp. 355–367).

## 16. Conclusions

Peripheral nerve stimulation represents a valuable addition to the perioperative analgesic armamentarium for oncologic surgical patients, supported by a growing, multi-layered evidence base spanning mechanistic investigations, oncologic series, robust RCTs in analogous surgical populations, and systematic reviews confirming sustained analgesic durability. The technology has evolved to enable minimally invasive, ultrasound-guided percutaneous placement of temporary 60-day systems that provide sustained, opioid-free analgesia without motor blockade, sensory deficit, or the commitment of permanent neuromodulation implantation. Oncologic-specific evidence from pilot studies and retrospective reviews confirms feasibility across diverse cancer pain subtypes—PTPS, PMPS, brachial plexopathy, and tumor-related neuropathic pain—with overall success rates of 58–67% [2] (pp. 118–125), [9], [10] (pp. 691–698), [11] (pp. S115–S191), [20] (pp. 819–826), [40] (pp. 3325–3348), [12] (pp. 191–203).

The opioid-sparing properties of PNS carry specific immunological significance in oncology, where perioperative opioid administration converges with surgical trauma and anesthetic effects to create a window of profound immunosuppression that may facilitate metastatic seeding. Although definitive prospective evidence linking PNS-mediated opioid reduction to improved cancer outcomes remains a priority research need, mechanistic data from NK cell function, macrophage polarization, and MOR-mediated tumor angiogenesis provide compelling biological rationale for perioperative opioid minimization. The evidence in this review is graded using the OCEBM 2011 framework, identifying the preponderance of Level 4 evidence in oncologic-specific applications and Level 1–2 evidence supporting the broader mechanistic and efficacy framework justifying extrapolation [8] (pp. 106–115), [10] (pp. 691–698), [11] (pp. S115–S191), [26] (pp. 3117–3139), [45] (pp. 342–348), [59] (pp. 644–649), [60] (pp. 305–310).

As perioperative oncologic medicine continues to evolve toward multimodal, ERAS-integrated care, PNS is positioned to play an increasingly central role—particularly given expanding regulatory clearances, growing payer coverage infrastructure, and the technical accessibility of contemporary ultrasound-guided percutaneous systems. The proposed clinical decision-making algorithm (Figure 1) is intended to translate the current evidence into a structured pathway for clinicians considering PNS in oncologic and cancer-related pain. Realizing the full clinical potential of PNS in cancer care depends on adequately powered, oncologic-specific RCTs, mechanistic biomarker investigations, CIPN-dedicated trials, and comparative neuromodulation effectiveness studies [29] (pp. 209–222), [56] (pp. 269–282).

## Figures and Tables

**Table 1:** Summary of key clinical and preclinical studies on peripheral nerve stimulation for perioperative and oncologic pain, graded by Oxford Centre for Evidence-Based Medicine (OCEBM) level of evidence.

Study	Design	OCEBM Level	Population	Key Finding
Vu, 2025 [12]	Scoping review (24 studies)	Level 4	Cancer-induced pain	PNS: NRS 8.29 → 3.04 (5.2-month f/u)
Harris, 2025 [13]	Systematic review (9 studies)	Level 1	Orthopedic surgery	tPNS safe; reduces pain & opioid use
D’Souza, 2025 [14]	Systematic review/meta-analysis	Level 1	Mixed chronic/postoperative	Sustained analgesia to 24 months
Lin, 2025 [15]	Systematic review/meta-analysis (8 RCTs)	Level 1	Lower-limb orthopedic	Marginal opioid reduction; no significant pain or ROM difference
Ilfeld, 2021 [16]	Multicenter RCT, sham-controlled	Level 2	Ambulatory orthopedic	Opioids 5 vs. 48 mg MME (days 1–7); pain 1.1 vs. 3.1
Goree, 2024 [17]	RCT, placebo-controlled	Level 2	TKA persistent pain	60% vs. 24% ≥50% responders (weeks 5–8); 54% vs. 26% mean pain reduction; 47% walk improvement
Gilmore, 2019, [18]	Multicenter randomized, placebo-controlled trial	Level 2	Post-amputation pain	58% vs. 14% ≥50% pain relief at weeks 1–4
Gabriel, 2019, [19]	Narrative review/feasibility summary	Level 5	Postoperative (mixed)	63% average pain reduction
Mainkar, 2020 [20]	Pilot case series	Level 4	Oncologic (n = 12)	7/12 (58.3%) success; PTPS, PMPS, PHN
Sudek E.W., 2024 [2]	Retrospective review	Level 4	Oncologic (n = 15)	10/15 (67%) successful across subtypes
Pingree, 2022, [21]	Cross-sectional observational	Level 4	Mixed chronic pain	73% success at 60 days; 61% maintained ≥3 months
Luna, 2025, [22]	Prospective observational cohort	Level 4	Mixed chronic pain	36.7% long-term response; depression predicts non-response
Engle, 2026, [23]	Multicenter randomized controlled trial	Level 2	Mixed chronic pain	85% responder rate; 67% pain reduction at 24 months
Mogedano-Cruz, 2025 [24]	Case report	Level 5	CIPN (oxaliplatin)	EORTC QLQ-CIPN20 improvement; maintained chemo dose
Sun, 2018 [25]	Preclinical	Level 5	Bone cancer pain	Arc/AMPAR mechanism; allodynia relief at 60 Hz
Manchikanti L., 2024, [11]	Evidence-based guideline	Level 1	Chronic pain	Level III evidence (moderate certainty) for 60-day PNS
Gill B., 2025 [26]	Evidence-based guideline	Level 1	Chronic pain	Consensus criteria for PNS candidacy and application

## Data Availability

No new data were created or analyzed in this study. Data sharing is not applicable to this article.

## Abbreviations

The following abbreviations are used in this manuscript: PNS, peripheral nerve stimulation; PENS, percutaneous electrical nerve stimulation; SCS, spinal cord stimulation; DRGS, dorsal root ganglion stimulation; ITDD, intrathecal drug delivery; ERAS, enhanced recovery after surgery; CIPN, chemotherapy-induced peripheral neuropathy; PTPS, post-thoracotomy pain syndrome; PMPS, post-mastectomy pain syndrome; NK, natural killer (cell); MOR, mu-opioid receptor; MME, morphine milligram equivalents; PGIC, patient global impression of change; NRS, numeric rating scale; RCT, randomized controlled trial; OCEBM, Oxford Centre for Evidence-Based Medicine; SANRA, Scale for the Assessment of Narrative Review Articles; PRISMA, Preferred Reporting Items for Systematic Reviews and Meta-Analyses.
